# Books and Pamphlets Received

**Published:** 1888-02

**Authors:** 


### BOOKS AND PAMPHLETS RECEIVED.

*Health and Home Library.* This is a new monthly gotten up to meet the popular demand for medical literature. The style, size and beauty of this Journal are certainly unexceptionable. Should the beauty and interest of its contents correspond with its exterior taste and embellishments, it will certainly become very popular. So far as the present issue is concerned it will please the popular reader rather than the educated physician. The management of such a periodical involves great moral responsibility on the part of those who conduct it, as the masses of men are easily swayed in wrong directions as touching the medical profession. The article in the present number against vaccination is at variance with the sentiment of the most enlightened members of the profession everywhere, and seeks through the exceptional cases and accidents to alarm the public mind and to make odious one of the grandest discoveries known to medical science.

*A Practical Treatise on Materia Medica and Therapeutics*, by Roberts Bartholow, M. A., M. D., LL. D., Professor of Materia Medica, General Therapeutics and Hygiene in the Jefferson Medical College of Philadelphia; Physician to the Philadelphia Hospital and Lecturer on Clinical Medicine on the same; Fellow in the College of Physicians; Member of the American Philosophical Society; Honorary member of the *Societie Medica Pratiques de Paris*, and of the Ohio, New York, Connecticut and Texas Medical Societies, and of the American Neurological Association; Author of Treatise on the Practice of Medicine; of a Treatise on Medical Electricity; of a Manual on Hypodermic Medication, etc., etc. Sixth edition revised and enlarged. New York: D. Appleton & Co. 1887.

In this new and revised edition of Bartholow's popular and excellent work on materia medica we find many changes and additions of interest and practical importance, the additions amounting to about one hundred pages, and we note that careful attention has been paid to the physiological action of remedies. This is well, for without it there can be expected little else than the same empiricism and guess work that has so largely prevailed in the past. We are pleased to note that the work has been adapted to the officinal standard, and that many recent improvements in the science and art of therapeutics have been added. We regard the book as second to none extant, in the department of materia medica and therapeutics.

*Druet's Surgeons' Vacdemecum;* A Manual of Modern Surgery. Edited by Stanley Boyd, M. B., B. S., London, F. R. S. C. England. Assistant Surgeon and Pathologist to the Charing Cross Hospital, and Surgeon to the Paddington Green Hospital for Children; Late Demonstrator of Anatomy in University College Hospital. Twelfth edition, with three hundred and seventy-three wood engravings. Philadelphia· Lea Brothers & Co. 1887.

This work, though called a manual, is a book of nine hundred and eighty-five large octavo pages, elegantly gotten up in calf, and profusely illustrated. Druet surgery has been so long and favorably known by the profession, both in England and America, that is scarcely necessary to speak of its merits. The work in old form was popular, but the present edition contains a thorough revision, bringing the same down to recent methods and improvements, so that it will constitute a very desirable work for both student and practitioner. In this last edition there are added no less than seventy-two new illustrations and many recent impovements in surgery. We recommend the book as amongst the very best and most practical now extant in the department of surgery.

*A Treatise on the Diseases of the Eye.* By Henry D. Noyes, A.M., M.D.' Professor of Ophthalmology and Otology in Bellevue Hospital Medical College; Surgeon to New York Eye and Ear Infirmary; President of the New York Ophthalmological Society, etc. New York: William Wood & Co. 27 Great Jones street. 1881. 360 pp. octavo.

The work has many illustrations, two of which are colored and very beautiful. The author has condensed into this work about all that is known of ophthalmic science. It is well and ably gotten up, and we commend it to the profession.

